# Women’s views on content and delivery methods for interventions to improve preconception health: a qualitative exploration

**DOI:** 10.3389/fpubh.2024.1303953

**Published:** 2024-02-21

**Authors:** Michael P. Daly, Ruth R. Kipping, James White, Julia Sanders

**Affiliations:** ^1^Department of Population Health Sciences, Bristol Medical School, University of Bristol, Bristol, United Kingdom; ^2^School of Medicine, Cardiff University, Cardiff, United Kingdom; ^3^School of Healthcare Sciences, Cardiff University, Cardiff, United Kingdom

**Keywords:** preconception care, preconception health, women’s health, intervention development, intervention design, qualitative, framework analysis, sequential exploratory design

## Abstract

**Background:**

Systematic review evidence suggests preconception health interventions may be effective in improving a range of outcomes. However, few studies have explored women’s views on potential content and delivery methods for these interventions.

**Methods:**

Participants were purposively sampled from respondents (*n* = 313) of a survey. Semi-structured, in-depth interviews were conducted to explore their views on seven candidate delivery methods for preconception health interventions: general practitioners (GPs), nurse practitioners, pharmacists, social media, personal texts and emails, pregnancy tests, and health education in schools. Data were analyzed using a data-driven framework analysis.

**Results:**

Twenty women were interviewed. Women wanted interventions to be easily accessible but allow them to conceal their pregnancy plans. They preferred to choose to receive preconception interventions but were receptive to health professionals raising preconception health during ‘relevant’ appointments such as contraceptive counseling and cervical smear tests. They wanted intervention content to provide trustworthy and positively framed information that highlights the benefits of good preconception health and avoids stigmatizing women for their weight and preconception actions. The inclusion of support for preconception mental health and the use of visual media, personalization, simple information, and interesting and unfamiliar facts were viewed favorably.

**Conclusion:**

Interventions to improve preconception health should reflect the sensitivities of pregnancy intentions, be easy to access in a way that enables discretion, and be designed to seek consent to receive the intervention. These interventions should ideally be tailored to their target populations and provide trustworthy information from reputable sources. The potential for unintended harmful effects should be explored.

## 1 Introduction

Worldwide, there are around 23 million miscarriages (1), 2.4 million neonatal deaths (2), and 260,000 neural tube defect-affected pregnancies each year (3). There is high- and moderate-certainty evidence that maternal exposures before conception, such as a lack of dietary folate, high body mass index (BMI), interpregnancy weight gain and physical inactivity increase the risk of these outcomes and others (4). However, most women do not begin folate supplementation before pregnancy (5), 32% of reproductive-age women are not sufficiently active (6), and, in high-income countries, one in two reproductive-age women have an overweight or obese BMI (7, 8). These risk factors are more prevalent among minoritized ethnic and socioeconomically deprived women (8–10). Limited systematic review evidence suggests preconception health interventions may be effective in reducing the risk of congenital anomalies and alcohol-exposed pregnancies and in improving infants’ birth weight and women’s diets, physical activity and weight loss (11, 12). National health organizations have called for further research on preconception health interventions, and recommend that this research includes the views of the public (13–15) to increase intervention success and government buy-in (16, 17).

Few studies have explored reproductive-age women’s views and experiences of preconception health interventions. Most previous studies have focused on preconception care delivered in healthcare settings (18–25), of which half have focused on women with diabetes (18–21). Women with diabetes have reported fears of being judged, labeled or lectured and encountering an unsupportive or authoritative healthcare provider to be barriers to their engagement with preconception care, and reassuring and approachable healthcare staff to be facilitators (18–20). They value receiving high-quality information (20, 21) and seeing a healthcare professional who knows and understands them and has dedicated time for preconception counseling (19, 21). Studies with women without chronic conditions have reported cost, accessibility issues (e.g., for rural residents) (22), a lack of time (20), and a reluctance to disclose pregnancy intentions (23, 24) as barriers to their engagement with preconception care. They have reported the reliability of information from a health professional and being able to see a familiar and respectful female professional as facilitators (23, 24).

An issue with the literature’s primary focus on preconception care delivery in healthcare settings is that healthcare providers have consistently highlighted low patient demand for preconception care and a lack of contact with women planning a pregnancy (26–28). ‘Broader strategies’ involving a wider spectrum of stakeholders and community-based methods may therefore be required to promote preconception health (29, 30). This approach is supported by the findings of our recent survey of 835 women in England (31). We found social media, texts and emails, health education in schools, and pregnancy test packaging to be highly acceptable methods for delivering preconception health interventions, as well as doctors, midwives and nurses. Similarly, women in a qualitative study in England (24) expressed that information about preconception health should be more widely available in the community, particularly in schools. A subsequent focus group study in Northern Ireland (25) reported that women and men generally favored non-medical sources of preconception health information, such as family and friends, the internet and social media.

A limitation of these previous qualitative studies was their use of convenience samples, where participants were opportunistically recruited via posters and university-wide emails (25) and pre-existing social groups (24). This meant their authors did not purposively recruit women representing a range of socio-demographic characteristics and attitudes toward candidate methods for preconception health interventions. Nor did they explore women’s views on content for these interventions. This qualitative study therefore aimed to explore reproductive-age women’s views on potential delivery methods and content for interventions to improve preconception health.

## 2 Materials and methods

### 2.1 Study design and researcher characteristics

This exploratory qualitative study constituted the second phase of a mixed methods project investigating acceptable preconception health intervention designs (31) and involved semi-structured, in-depth interviews with women aged 18–48 years. The study was undertaken from a position of critical realism (32). Data were iteratively collected and analyzed using a data-driven, framework analysis approach (33), which complemented this position (34). The study received ethical approval from the South West – Frenchay Research Ethics Committee before its conduct (19/SW/0235) and is reported following Standards for Reporting Qualitative Research guidelines (35). The study team comprised investigators with backgrounds in public health research and practice, epidemiology, psychology and midwifery. All had prior experience of qualitative research.

### 2.2 Study population and participant selection

#### 2.2.1 Eligibility criteria

Participants were eligible for inclusion (Table 1) if they participated in our prior survey of women registered with seven general practices in the West of England (31), and expressed interest in being interviewed about their responses. Further information on the survey’s sampling methods is available elsewhere (31).

**Table 1:** Eligibility criteria for the preceding survey study.

Inclusion criteria	1. Registered as a patient with a participating general practice
	2. Female
	3. Aged 18 to 48 years
	4. Main spoken language coded as English
Exclusion criteria	1. Current pregnancy known to the patient’s general practice
	2. Having a condition causing permanent infertility
	3. Having an enduring condition involving profound cognitive impairment
	4. Likely to be distressed by pregnancy-related content (e.g., pregnancy loss at any stage in the last 3 months, perinatal mortality ever)

#### 2.2.2 Participant sampling

Of the 835 survey participants, 313 (37.5%) expressed interest in being interviewed. To promote diversity between participants (36) and enhance the transferability of our findings (37), we used a maximum variation purposive sampling approach (38). Our sampling criteria (39) (Supplementary Material S1) were informed by our survey findings (31). The strongest correlates of low knowledge and less positive attitudes toward preconception health were younger age, lower household income and nulligravidity; we aimed to achieve an approximately even split for these criteria. We monitored secondary criteria while recruiting participants to ensure diversity in their coverage (39). These included: ethnicity; country of birth; pregnancy intentions (including women who were ambivalent about or did not want a future pregnancy); attitudes toward preconception health; and acceptability ratings for the seven intervention delivery methods explored in this study.

#### 2.2.3 Participant recruitment

Invitation emails were sent with the study’s participant information sheet (PIS) and consent form to 46 women. We aimed to include approximately 10 participants in each grouping of our dichotomous primary criteria (20 overall), informed by guidance that datasets involving 10 interviews or fewer are conducive to theme development where participant groups are homogenous (37). However, we continuously assessed the dataset during data collection to ensure the final sample was sufficient to justify claims of patterned meaning, validity and information power (37, 40).

### 2.3 Data collection

#### 2.3.1 Interview schedule

We used in-depth, semi-structured interviews to support participants to freely express their views, reduce the risk of socially desirable responding, and facilitate probing of participants’ responses (41–43). We developed an interview topic guide (Supplementary Material S2) informed by our survey findings (31), a review of the literature (18–25) and through consulting academic researchers. The topic guide included questions to elicit participants’ views on seven candidate delivery options for preconception health interventions, found to be acceptable to women in our survey (31), and acceptable and appealing content for these interventions. The options were: general practitioners (GPs), nurse practitioners, pharmacists, social media, personal texts and emails, pregnancy tests, and health education in schools. MD conducted pilot interviews with five women, approached as peers or via a local public involvement network. Commonly misunderstood phrasings were re-worded. The topic guide was refined as new areas of interest emerged from the interviews.

#### 2.3.2 Interviews

Interviews (*N* = 20; September–December 2021) lasted an average of 57 min (range: 37–79 min, reflecting the diversity in views and experiences). All participants chose a telephone interview. MD conducted 19 (95%) interviews. JS interviewed the final participant, who requested a female interviewer. Interviews were audio-recorded using an encrypted audio-recorder after consent was confirmed. Participants received a £20 shopping voucher to thank them for participating (44).

### 2.4 Ethical considerations

Participants provided consent for the publication and sharing of anonymized information. We anticipated that the topic of pregnancy might induce distress in participants who had experienced adverse pregnancy outcomes or infertility. We thus stated in the PIS and interview pre-briefing that participants could skip questions, stop the interview and/or withdraw from the study at any point.

### 2.5 Data analysis

Data were analyzed according to the framework method (33). Interviews were transcribed verbatim, transcripts were anonymized and imported into the NVivo 11 software package, and familiarization was undertaken (33). Deductive code labels (Supplementary Material S3) were applied to all relevant excerpts (45), as were inductive codes developed from the first three transcripts (coded in duplicate by the research team) (33) if these were agreed to be sufficiently relevant to the research questions. All investigators agreed on an initial analytical framework to index the remaining transcripts, and discussed potential refinements (33). The data were charted into matrices using Microsoft Excel, with each matrix representing an intervention delivery method (33). Data for each code were paraphrased and added to the corresponding participant’s matrix cell with reference to salient quotations (33). Candidate themes were developed by identifying data patterns within and between matrices. Themes were chosen based on prevalence, the study’s aims and deductive codes, and inductively-derived concepts, and elaborated through analytical memos (33). The transcripts were reviewed to confirm candidate themes formed a ‘coherent narrative’ of the data and addressed the research questions (37). The final themes and subthemes were written up as an analytic narrative (45).

### 2.6 Data quality

As rigor and quality are considered requisite considerations for framework analysis (33), we incorporated the concept of data trustworthiness, a theorisation of data quality developed for thematic analysis (46). Supplementary Material S4 outlines our quality-assurance measures for each data trustworthiness criterion.

## 3 Results

### 3.1 Participant characteristics

Twenty women took part (43.5% of invitees). Table 2 shows there was an even split of gravid and nulligravid participants and 18–29 and 30–48-year-old participants. Half (45%) of participants had experienced at least one live birth and a fifth (20%) had experienced a miscarriage. Two-thirds (65%) reported a desire for a future pregnancy. Almost half (45%) had household incomes below £32,000, compared with 43% of the UK population (47). The proportion of participants who were UK-born was similar to the national average (85% vs. 84.3%) (48). A greater proportion of participants had a minority ethnicity (25% vs. 15.2%) (49) and were university graduates (65% vs. 42%) (50) than the national average. We recruited participants with a range of acceptability ratings for the discussed intervention delivery methods (Table 3). There was a broadly even split of participants with ‘low’ knowledge of preconception health (*n* = 9; listed ≤2 of the preconception risk factors assessed in our survey (31)) and ‘high’ knowledge (*n* = 11; listed ≥3 assessed risk factors). We recruited participants with a range of attitudes toward preconception health (Supplementary Material S5).

**Table 2:** Characteristics of the interview participants*.

Variable	Response categories	Study sample	
		*N*	(%)
Age (years)	18–19	1	5
	20–24	2	10
	25–29	7	35
	30–34	4	20
	35–39	5	25
	45–48	1	5
Household income (£)	Less than 13,000	1	5
	13,000–18,999	2	10
	19,000–25,999	1	5
	26,000–31,999	5	25
	32,000–47,999	4	20
	48,000–63,999	1	5
	64,000–95,999	2	10
	More than 96,000	4	20
Ethnicity	White	15	75
	Mixed/Multiple ethnic groups	1	5
	Asian/Asian British	1	5
	Black/African/Caribbean/Black British	2	10
	Other ethnic group	1	5
Education	University	11	55
	Intermediate	4	20
	Secondary school	4	20
	Still in education	1	5
Country of birth	The UK	17	85
	Other†	3	15
Gravidity	Previously pregnant	10	50
	Never pregnant	10	50
Livebirth(s)	Yes	9	45
	No	11	55
Adverse pregnancy outcome(s)	Yes ‡	4	20
	No	16	80
Previous infertility	Yes §	5	25
	No	15	75
Pregnancy desire	Currently trying to become pregnant	3	15
	Would like to get pregnant in the next 1–2 years	3	15
	Would like to get pregnant in the next 3+ years	7	35
	Not sure/Do not know	6	30
	Would definitely not like to get pregnant	1	5

*The data in this table correspond to the interview participants’ questionnaire responses in our prior survey study (31). †African and South American countries. ‡All four participants reported a miscarriage. §Participants who were unable to become pregnant after ≥ 12 months of trying and/or who had ever sought medical or professional help for infertility.

**Table 3:** Participant acceptability ratings for each discussed intervention delivery method*.

	Very acceptable	Somewhat acceptable	Neither acceptable nor unacceptable	Somewhat unacceptable	Very unacceptable
GP	12 (60%)	2 (10%)	1 (5%)	2 (10%)	3 (15%)
Nurse	11 (55%)	4 (20%)	1 (5%)	2 (10%)	2 (10%)
Pharmacist	2 (10%)	5 (25%)	8 (40%)	2 (10%)	3 (15%)
Social media	13 (65%)	5 (25%)	0 (0%)	1 (5%)	1 (5%)
Personal text/email †	13 (65%)	4 (20%)	0 (0%)	2 (10%)	1 (5%)
Pregnancy test	16 (80%)	3 (15%)	0 (0%)	0 (0%)	1 (5%)
Health education in schools	15 (75%)	2 (10%)	1 (5%)	1 (5%)	1 (5%)

*The data in this table correspond to the interviewed participants’ questionnaire responses in our prior survey study (31). †E.g., from one’s general practice.

### 3.2 Themes

The themes and sub-themes developed from the data are shown in Table 4; these are the focus of the analytic narrative presented below. The specific points participants made about each of the seven intervention delivery methods are presented in Supplementary Material S6.

**Table 4:** Thematic map of study findings.

Major theme	Sub-themes (with main points)
Theme 1: intervention delivery	1.1 Accessibility Convenience of texts, emails and social media versus the relative inaccessibility of primary care practitioners Pharmacists and practice nurses generally considered to have better availability than general practitioners (GPs) 1.2 Discretion Pregnancy planning considered “*quite a private business*” Texts, emails, social media and pregnancy tests considered discreet mediums Health professional confidentiality considered crucial 1.3 Consent to receive interventions Choice-enabling suggestions included opt-in options and content warnings Considered more acceptable for a health professional to raise preconception health in consultations relating to reproductive issues or women’s health 1.4 Trustworthiness Need for reputable ways of delivering information online (e.g., ‘.org’ websites, professional moderators) Trust in advice from health professionals depends on their perceived skill set and knowledge remit 1.5 Positively-framed risk communication Suggestions for acceptable preconception risk communication included highlighting the benefits of good preconception health, emphasizing that adhering to guidelines does not ensure a healthy baby, and avoiding stigma
Theme 2: intervention content	2.1 Desirable content Desirable content included a focus on mental health, self-care and where to find support, the why and how of preconception recommendations, and tailored content 2.2 Unacceptable content (weight-centrism) A few participants expressed strong views that weight- or calorie-based targets would deter them from engaging in preconception interventions and that health professionals are overfocused on advising weight loss 2.3 Appealing content Appeal-enhancing suggestions included eye-catching visual media, easy-to-read and to-the-point information, and interesting or surprising facts 2.4 The power of personalization Personal touches were frequently described as a way of enhancing appeal Examples included highlighting the personal relevance of provided information and using real people’s lived experiences as examples

#### 3.2.1 Theme 1: intervention delivery

##### 3.2.1.1 Accessibility

Participants commonly highlighted the importance of accessible interventions. For instance, a perceived advantage of sending information by personal text or email was the convenience for recipients:

*“I think it’s brilliant… He’s [GP] sent me the information [by text/email] and I’ve got it there in front of me within minutes. Instead of me having to go up to the surgery...”* (p. 04, 30–34 years, previously pregnant).

Participants also spoke about how “*accessible*” (p. 13) information provided through social media can be and how passive, unintentional information receipt can often lead to real engagement:

*“[Social media] is one of the best ways you can communicate with people. Because it comes up accidentally. People get really bored on their phones, so they click on things they do not really want to click on, but they end up reading and engaging in.”* (p. 03, 25–29 years, nulligravid).

Conversely, primary care practitioners were widely perceived to be relatively inaccessible. Participants reported that they only contact these professionals about illnesses and medical emergencies, as they lack the time to merely provide “*information*” about issues like preconception health:

*“…we tend to avoid contacting the GP if you are not unwell because you feel like you are wasting a doctor’s time, that they could be looking after people that are actually unwell… Whereas this is more: ‘I just need a chat and information’.”* (p. 01, 35–39 years, previously pregnant).

The brevity of primary care appointments was also considered a barrier to exploring issues in depth:

*“…the appointment times are just not long enough and I do prefer to read more deeply around different things.”* (p. 18, 25–29 years, nulligravid).

This perceived lack of availability was particularly apparent in discussions about general practitioners (GPs). Participants expressed that they would be willing to discuss their preconception health with GPs if there was “*an opportunity*” (p. 02). However, they felt it’s “*very noticeable*” that GPs do not have “*very much time*” (p. 03), meaning “*you do not feel like you can have a proper conversation*” (p. 03) and it’s not “*appropriate”* to ask GPs questions such as *“‘shall I take this vitamin?*’”(p. 10). Pharmacists were considered to have better availability than GPs. Nurses were also generally considered to have “*more time”* (p. 03) and be “*a lot easier to see*” (p. 09) than a GP. It was thus felt that topics such as preconception health can be raised in a nurse consultation, alongside other care:

*“…I could bring that up in a blood test or my flu jab… to me, there’s a lot more downtime in a nurse’s appointment than there is in a GP [appointment].”* (p. 09, 20–24 years, nulligravid).

Finally, participants commonly demonstrated a desire to avoid adding additional burden to the national health service (NHS) and for access to “*services that specialize in women’s health”* (p. 08) to be improved:

*“With the NHS being so under-resourced... it’s what we are always encouraged to do, isn’t it? Minor things, go to a pharmacy first.”* (p. 10, 20–24 years, nulligravid).

##### 3.2.1.2 Discretion

Participants considered pregnancy planning to be “*quite a private business”* (p. 12) and felt that *“a lot of women would not necessarily tell anybody they were going to try for a baby*” (p. 19). Providing preconception health information and services discreetly was thus commonly considered advantageous or necessary. For instance, personal texts and emails were viewed as “*a nice and discreet way*” (p. 18) to provide information, websites were considered “a *good resource*” that “*anyone can access in the privacy of their own homes*” (p. 19), and information in pregnancy test packaging was considered “*quite private*” (p. 13).

The importance of discretion was also evident in suggestions for enhancing the acceptability of interventions. For instance, participant 18 favored placing information about preconception health “*inside the packaging*” of pregnancy tests over a vendor “*loudly waving a leaflet and talking about pregnancy.”* Participant 05 emphasized the importance of preconception health information provided through social media being “*visible just to you*” and was reluctant to “*respond to something on social media about pre-pregnancy*” because ‘*basically anyone could see… and pin it back to you*.” The importance of providing information to school pupils that they can access discreetly was also highlighted:

*“Leaflets in school are good but not great because everyone can see you picking it up… more of an online resource is best.”* (p. 10, 20–24 years, nulligravid).

Discretion was also a consideration for methods involving health professionals. Unlike the above examples, this tended to relate more to the importance of confidentiality, highlighting a need to consider the many forms of discretion. For instance, a perceived advantage of discussing preconception health with a GP was that *they are bound “by* *various confidentiality rules”* (p. 16) and can be trusted not to share personal information:

*“I think the GP… for a lot of people, is a safe person. Somebody who is a healthcare professional, somebody they trust.”* (p. 19, 45–48 years, previously pregnant).

Some divergent views on this were noted, including the security of GP medical records and confidentiality concerns for patients with family or friends working “*within the GP practice”* (p. 08). Participants commonly reported that enabling privacy may be an issue if pharmacists were used as an intervention medium. This mainly related to the public nature of discussions with a pharmacist:

*“…there would probably be certain things I would not want to just discuss [with a pharmacist] when potentially someone could just walk through the door.”* (p. 13, 18–19 years, nulligravid).

Participants reported that they might therefore “*shut the conversation down*” (p. 08) if a pharmacist attempted to raise the topic of preconception health. However, some felt this would be more acceptable if pharmacists had “*time to take someone aside*” (p. 03) and consult them in a “*private room*” (p. 19), and allowed this private consultation to be anonymous and booked discreetly online:

*“If you could book a private appointment online so you do not have to physically go in and physically ask for a private session… then it’s no different than just booking a doctor’s appointment.”* (p. 12, 35–39 years, nulligravid).

*“You could go to any pharmacy if you did not want to speak to someone that may know you… if you wanted to, give a false name.”* (p. 05, 35–39 years, previously pregnant).

##### 3.2.1.3 Consent to receive interventions

Participants expressed mixed views on whether individuals need to consent to receive preconception health interventions. Some felt this wasn’t necessary, as it’s “*definitely something that women need to know*” (p. 06) and recipients can “*ignore*” (p. 08) provided information. However, others felt it would be an issue if recipients “*have not chosen to see*” (p. 10) this information as it might feel “*intrusive*” and “*triggering”* (p. 19) for some, such as those who have “*just had a miscarriage”* (p. 10), and that people should therefore be given *“the choice*” (p. 15). Suggested methods for enabling this choice included a scoping message asking “*are you happy to receive emails on this topic?”* (p. 13), a “*click to find out more*” (p. 14) or “*opt-in*” (p. 03) option, and asking patients “*is it okay if I send you this stuff?*” (p. 13) in healthcare appointments. Participants also suggested having a content warning, for example at the “*very top of the email*” (p. 05), so that recipients can make an informed decision about whether to delete or read it. A further choice-enabling suggestion for pregnancy test packaging was to provide leaflets *by* rather than *in* the boxes and use these leaflets to signpost to “*where [one] can look*” (p. 17) for information. This contradicted the suggestion for a leaflet to be inside the box to enable discretion.

A perceived benefit of social media as an intervention medium was that people choose to use social media and have some control over the content they see and engage with. Participants therefore felt this method is not as invasive as having “*information sent straight to your phone*” (p. 10):

*“…it [social media] never feels imposing… you can just choose to scroll past something if you are not comfortable with it.”* (p. 13, 18–19 years, nulligravid).

Some participants also considered that targeted social media advertisements would be acceptable as these tend to relate to relevant online *“searches or conversations*” (p. 01), social media algorithms are “*very for that person*” (p. 09), and you can choose not to see similar advertisements again:

*“Targeting people with specific ads if they are in the right age group or they have thought about pregnancy before would be good… if it affects you… you can say that you do not want to see the ad anymore.”* (p. 14, 25–29 years, nulligravid).

Others disagreed with this view. They felt the use of targeted social media advertisements would be “*invading people’s privacy if they have had bad pregnancy experiences*” (p. 18), and that the imperfection of algorithms means there would be targeting issues. They thus felt it would only be appropriate to provide this information to those who had explicitly sought it out:

*“You might have spoken about pregnancy tests and started getting things about children and how to conceive, when actually that’s the opposite.”* (p. 08, 25–29 years, nulligravid).

Some participants also felt that health professionals raising the topic of preconception health would be inappropriate as this might be seen as disregarding the wishes of women who cannot have or do not want children and “*panic*” those who “*do not have all the time in the world”* (p. 11) left to become pregnant. Conversely, other participants felt it would be acceptable for health practitioners to initiate a conversation about preconception health, and that questions like “*Do you feel well equipped with the knowledge to have a successful pregnancy?*” should be “*standard*” (p. 03). In support of this view, participants argued that “*a lot of people will not open up*” or “*want to admit that they do not know*” (p. 03) this information. It was also argued that there are “*so many missed opportunities*” (p. 03) for this information to be provided to patients and “*there needs to be a way”* to know that GPs *“offer these kinds of services*” (p. 05). Some participants suggested that it would be more acceptable for a health professional to raise these topics in consultations relating to reproductive issues or women’s health, such as contraception counseling and cervical smear tests:

*“…nobody ever says to me [during smear tests]: ‘And are you trying for a baby?’… that would be an ideal time”* (p. 12, 35–39 years, nulligravid).

Others felt that providing preconception health information would be more appropriate if the recipient was purchasing or had mentioned a product (e.g., folate supplements) or issue related to pregnancy, or had indicated their pregnancy intentions:

*“… if there was a setting on there [the NHS app] to say that ‘yes, I want a child at some point in my life’…. I think that setting would then give GPs the option to bring it up.”* (p. 09, 20–24 years, nulligravid).

Participants also suggested ways that healthcare service providers could advertise preconception health information and services so that patients can choose whether they engage with these. These suggestions included “*a welcome pack*” (p. 05) for new registrations and other promotional materials, such as posters, webpages and leaflets inserted into shopping bags by pharmacists when selling related products such as “*ovulation tests, pregnancy tests”* (p. 08):

*“Even if you just stuck it on the back of folic acid, as a: ‘go to this website’… You just need to raise the awareness of where to go...”* (p. 17, 35–39 years, previously pregnant).

##### 3.2.1.4 Trustworthiness

Participants commonly spoke of the importance of trustworthy information and reputable sources. For instance, participants frequently highlighted the considerable amount of poor-quality information available through social media and the internet, which can be “*hard to filter through*” (p. 16) to find legitimate information. Some participants therefore felt that communicating preconception health information through social media would be problematic as it may get *“lost amongst all of the reams of misinformation*” (p. 18) and “*with pregnancy, you have got to be so careful*” (p. 06). Others felt this highlighted a need for using “*reputable ways”* (p. 03) of delivering preconception health advice on social media and the internet. These included partnerships between trusted institutions and subject experts and the use of ‘.org’ websites (p. 07), peer-reviewed scientific literature (p. 18), professional moderators (p. 15), and recommendations from reputable health organizations:

*“They [period tracking app] go by NHS guidelines and WHO [World Health Organization] and all that kind of stuff. So they are a really good source of information.”* (p. 03, 25–29 years, nulligravid).

Some participants favored advice from a recognized health professional over receiving information online. They felt that with health professionals, you can be more sure you have “*got the right information*” (p. 06) and that they would not make a health behavior change “*unless a health professional tells [them] to*” (p. 10). However, many participants expressed that their trust in a health professional’s advice would depend on their perceived skill set and knowledge remit. For instance, participants commonly saw pharmacists as the “*medicine person*” (p. 08) with whom they would feel comfortable discussing preconception supplements and the potential impact of “*any other medication*” (p. 01). While some said they would speak to pharmacists about “*any kind of [preconception] topics*” (p. 07), others stated they would not seek or trust their advice on more general “*lifestyle changes”* (p. 09). Some felt it would only be acceptable for a pharmacist to initiate a discussion about preconception health “*if they are trained*” to (p. 06) or if their pharmacy “*had a specific interest in maternal health*” (p. 19).

This mix of views was also observed for GPs. Some participants expressed a reluctance to discuss “*lifestyle changes*” with a GP (p. 06). This was associated with a view that GPs’ knowledge is “*spread over so many things*” (p. 17) and that someone else who is “*very specialized”* (p. 13) in reproductive health might know more. A contrasting view was that GPs are “*the real medical practitioners*” (p. 02) who *“give you the facts that you can trust*” (p. 10). Nurse practitioners were generally considered to have a more practical skillset. For some, this meant that nurses are a less acceptable source of guidance:

*“Some nurses have a very, very, very low knowledge of nutrition and they are more practical. So they are more wound care.”* (p. 03, 25–29 years, nulligravid).

Others felt this practical skillset means that nurses often have more “*hands-on experience of how things have affected people”* (p. 10) and are “*a bit more down-to-earth*” (p. 12) than GPs. Some participants felt that “*the more appropriate person*” would be “*whoever is more of a specialist on the topic”* (p. 18) of preconception health.

##### 3.2.1.5 Positively-framed risk communication

Participants suggested several ways to enhance the acceptability of information about preconception risk factors. One suggestion was to emphasize that adhering to all preconception health guidelines does not guarantee a healthy baby, to minimize the potential for people to “*think that they are the problem*” (p. 09) and self-blame for adverse outcomes. A further suggestion was to strike “*the right balance”* (p. 13) between acknowledging the different emotions women may feel toward pregnancy and ensuring the provided information is factual and non-euphemistic:

*“You do not want to dress up the facts so much that they get lost…it’s information that you want to deliver quite softly but still factually.”* (p. 13, 18–19 years, nulligravid).

Some participants also spoke of the importance of positive framing and highlighting the benefits of good preconception health rather than focusing on risks, harms and actions to avoid (i.e., “*‘do not do this, this is the risk’”* (p. 12)). These participants felt this approach is more effective and memorable, and that people “*like aiming for the positives”* (p. 17):

*“…when I was smoking, it made absolutely zero difference to me… that doom and gloom stuff… I’ve responded a lot better to stuff that’s been much more positive.”* (p. 12, 35–39 years, nulligravid).

Participants also felt a risk-averse, “*better to be safe than sorry*” (p. 20) approach to health messaging may be desirable as people’s risk perceptions vary greatly, and that people are more likely to disregard information that relates to risk rather than guaranteed health outcomes:

*“If you said to someone: ‘If you exercise, you’ll have an extremely healthy pregnancy’… everyone would be exercising. But because that’s not true, it’s just: ‘Yes, you are going to be reducing any potential health risks’… Do people really understand what that means?”* (p. 03, 25–29 years, nulligravid).

Further, some expressed that people should not be judged for their preconception actions or pressured into changing their behavior, as this is unacceptable and unlikely to affect change. Preferred strategies included acknowledging that behavior changes are “*difficult for everyone”* (p. 05), accepting people’s circumstances, and focusing on risk mitigation:

*“The work that I’ve seen done best is about… harm reduction. ‘Okay, we accept where you are, how can we help you to minimize risk?’… I do not think the other way of doing it, of just saying: ‘Oh, well, just stop’ makes a difference.”* (p. 12, 35–39 years, nulligravid).

Participant 17 also alluded to the importance of respecting the reproductive choices of women whose health conditions are associated with greater risk of adverse outcomes. This would mean tailoring messages to ensure acceptable risk communication for these women:

*“….[At a discussion] someone had said that women with diseases should not reproduce, which was a bit harsh to take in… I think there can be an outcome, but I’ve actually seen quite a lot of…. [people] with diseases like epilepsy, like diabetes… and the babies have been fine.”* (p. 17, 35–39 years, previously pregnant).

The importance of acceptance was also evoked in participants’ views on GPs as potential interventionists. Some participants expressed that they would *“feel comfortable discussing basically anything with a GP because it feels confidential and without judgement”* (p. 13). Others highlighted that they would be comfortable discussing preconception health with a GP “*as long as they keep their personal opinions to themselves*” (p. 10). Participant 16 described an experience where she considered not telling her GP about skiing while pregnant because she “*knew that they’d not really approve*.”

#### 3.2.2 Theme 2: intervention content

##### 3.2.2.1 Desirable content

Participants highlighted a range of content they felt should be included in preconception health interventions. This commonly included “*general advice*” (p. 02) on “*what to do if you want to be pregnant*” (p. 09) and preconception “*changes”* (p. 05) to consider. Others suggested this guidance should include the specifics of recommended preconception practices, such as the exact “*dosage*” (p. 17) of folic acid and safe alcohol consumption limits, so that women can make decisions “*with a bit more knowledge behind them*” (p. 20).

Mental health was highlighted by some as an important focus for preconception health interventions. Participants felt that factors like “*stress*” are a “*big problem*” (p. 04) for outcomes such as miscarriage and that the importance of people’s mental preparedness for pregnancy is typically overlooked, despite mental health being *“just as important as physical health”* (p. 04):

*“It’s not so much of a main focus when you are trying to have a baby… [that] you and your mental [health] are ready.”* (p. 09, 20–24 years, nulligravid).

Some participants expressed that they would be more interested in preconception health interventions if they included a focus on “*self-care*” (p. 09) and signposted to where people *“can find support*” (p. 14), including support for individuals *“struggling to get pregnant*” (p. 14). A perceived advantage of social media as a delivery method was its ability to help users to *“find support with other women”* (p. 09).

Participants also suggested that intervention content should be tailored to its target population, particularly with regard to age. All participants agreed that providing at least *some* information about preconception health through schools would be appropriate. Specifically, participants suggested providing pupils with “*some really basic detail”* that *“links with good health advice”* (p. 12) but includes something specific to preconception health. They also suggested making pupils aware of relevant services and information sources, and highlighting the preconception period as a unique phase of the pregnancy journey:

*“…breaking it down into the different phases… living a normal life, then the preconception phase, then you are pregnant but you are not necessarily aware of it, and then you have got the trimesters of pregnancy… building up that awareness that it’s not just as simple as not pregnant, pregnant.*” (p. 16, 30–34 years, previously pregnant).

Participant 04 also spoke of the importance of highlighting the “*problems that can occur*” in the preconception period, including infertility, as this “*should not be a taboo subject*.” She felt this had not been communicated in her education at school, which resulted in a conflict between her reproductive expectations and reality:

*“I assumed that by the age of twenty-eight, I would have two children… I’m now [in my early thirties] and I’ve not got none... I do not think that’s made clear enough, about infertility...”* (p. 04, 30–34 years, previously pregnant).

Some participants felt information about preconception health should be communicated to pupils, particularly older teenagers, in the same way it would to adults, as “*making everything very kiddy and funky”* (p. 06) can be off-putting and impair understanding:

*“…being factual and realistic can just be the best way of delivering things to kids, especially when you are talking about sixteen-year-olds.”* (p. 06, 35–39 years, previously pregnant).

Others felt it may be better for the focus of this information to be “*women’s health*” (p. 12) or the importance of preconception health more generally, as topics like “*babies*” (p. 12) and pregnancy may be off-putting or of little interest to this cohort:

*“Just give them: ‘Okay, this is important… to have good pre-pregnancy health for these reasons’. Just general. Not being too specific. Because they are sixteen, I do not think they want to get pregnant.”* (p. 07, 25–29 years, nulligravid).

Participants who felt younger children should be given some level of information about preconception health noted that “*a fun way*” (p. 09) of relaying this information to this age group may be required:

*“…almost like a game, like ‘oh, this woman, or this person, wants to have a baby… in six months. What should they be doing to make sure that their pregnancy is as healthy as can be?’.”* (p. 09, 20–24 years, nulligravid).

##### 3.2.2.2 Unacceptable content (weight-centrism)

A few participants expressed strong views that it’s important for preconception health interventions to “*not be weight-centric*” or even “*mention weight*” (p. 09). These participants expressed that repeatedly being advised to lose weight “*gets to you, massively*” (p. 04) and that the possibility that a healthcare provider might “*mention anything about weight*” would make them “*anxious*” (p. 09). Participant 09 (20–24 years, nulligravid) felt body weight is not a “*fair determinant of health*,” that body mass index (BMI) is “*outdated, sexist*” (p. 09), and that there’s *“so much pressure on women… to fit a certain box of ideals”* (p. 09). She described how she was “*stepping away*” from having *“so much of [her] medical care and [her] life revolve around”* her weight and that weight-, BMI- or calorie-based targets “*would a hundred per cent”* deter her from engaging with preconception health interventions. Conversely, she expressed that she would be interested in interventions that focused on *“healthy habits”* such as finding a way to be physically active that *“makes your body feel good*” or making meals more “*nutritional*.” Participant 04 described prior expectations from health professionals for her to lose weight as “*ridiculous*,” and the way she had been spoken to about this as “*awful*.” She felt health professionals are overly focused on advising weight loss and lack the ability to support patients beyond this:

*“They just tell you to lose weight…. But if you cannot lose weight, what else can you do?... they do not seem to be able to help you.”* (p. 04, 30–34 years, previously pregnant).

##### 3.2.2.3 Appealing content

Participants expressed a range of views on whether and how to maximize the appeal of intervention content delivered through the discussed intervention mediums. Some felt appeal-enhancing strategies are not needed, as women wishing to become pregnant will already be interested in this topic:

*“It’s [pregnancy] not something that you do by invitation. It’s more like something you have already planned and the information just happens to be there to help you along the way. Rather than it being something that entices you.”* (p. 01, 35–39 years, previously pregnant).

Participant 08 expanded on this view by saying that appeal may only be needed for people who have ambivalent pregnancy intentions:

*“…[for people wishing to become pregnant]a simple leaflet would do. But if it was a resource for women that were maybe on the fence then, yeah, it might be.”* (p. 08, 25–29 years, nulligravid).

A multitude of methods for enhancing intervention appeal were suggested by participants who felt this could be beneficial. These included the use of “*eye-catching*” (p. 10) visual media involving images, video and color. Participants expressed that this media can turn *“a very boring, factual [social media] post into something that catches people’s attention”* (p. 13) and that it’s *“almost not even worth”* (p. 03) putting information on social media without it:

“*…all the communication [on social media], pretty much, has to be through a really quick video or graphics that lead to information that is simple to digest… You always want that stimulation first.*” (p. 03, 25–29 years, nulligravid).

Participants also highlighted the importance of “*simple*” (p. 08), “*easy-to-read*” (p. 17), and “*to the point*” (p. 04) information that is free from “*medical jargon*” (p. 08), as information that is “*really complicated… puts people off*” (p. 19):

*“You don’t want a video that’s five minutes long, right? It has to be appealing and short.”* (p. 07, 25–29 years, nulligravid).

However, some suggested that this initial, brief information could be supplemented with signposting to “*more complex bits of information”* (p. 03) for people who “*want to read more”* (p. 19). The use of interesting or “*surprising*” facts was another suggested way of ensuring recipients “*want to find out more*” (p. 03). Participants also described that appeal can be enhanced by ensuring the provided information has clear *“themes and a narrative”* (p. 03) and explains the why and how of preconception recommendations:

“*I really like to know how things work. So why am I going to be taking the folic acid? How is that going to be helping me?*” (p. 09, 20–24 years, nulligravid).

##### 3.2.2.4 The power of personalization

Participants frequently described the use of personal touches as a way of enhancing the appeal of preconception health interventions. This included suggestions to offer or signpost to more individualized information and making the provided information “*more personal*” (p. 15) through, for instance, using a patient’s name, following on from a relevant conversation, or tailoring information to the recipient’s personal risk factors. Highlighting the personal relevance of preconception health information was another suggested way of enhancing engagement:

*“It could even be a very transparent leaflet saying: ‘a lot of women… [of] childbearing age have reduced knowledge of nutritional interventions and exercise interventions’… I think people are more inclined to know more once they think: ‘Oh, I’m in that category…’”* (p. 03, 25–29 years, nulligravid).

Participants also valued the “*personal touch*” (p. 12) of a health professional-initiated conversation, as they “*would not pay any attention to”* a leaflet (p. 12), and expressed that they would be more comfortable having this conversation with a familiar professional:

*“I do not want to share something that’s quite so personal with someone I do not know.”* (p. 17, 35–39 years, previously pregnant).

Finally, participants widely considered real people’s lived experiences to be a more “*personal*” (p. 09), “*relatable*” (p. 03) and memorable way of communicating health information than “*textbook*” (p. 02), scientific information:

*“… if I read someone’s experience, I would take that into account more than just information, information, information.”* (p. 04, 30–34 years, previously pregnant).

## 4 Discussion

National health organizations such as England’s Department of Health and Social Care have called for more research with women to explore how best to engage them in preconception health interventions (13–15). This is the first qualitative study to explore views on content and delivery methods for preconception health interventions delivered in both healthcare- and community-based settings, with purposively-recruited women to capture a variety of experiences and perspectives. To increase appeal and acceptability, women suggested preconception interventions should ensure privacy, sensitivity, easy access and appropriate consent, and provide positively-framed information that highlights the benefits of good preconception health. They favored the use of visual media, ‘trustworthy’ information from reputable sources, interesting and unfamiliar facts, simple information with signposting to more complex information, and the use of personalized information.

### 4.1 Integration with prior research

Participants felt individuals should choose to receive preconception health interventions, but that it may be acceptable for health professionals to raise preconception health with patients. One of the justifications for this was that this may address the ‘missed opportunities’ for improving preconception health (14, 51). This opportunistic delivery was considered more acceptable in encounters relating to reproductive issues or women’s health, such as cervical smear tests and contraceptive counseling. This view was also identified in Tuomainen et al.’s (24) qualitative study of 41 women; collectively these findings support policy recommendations that healthcare services should meet women’s full range of reproductive health needs at the same time and place (14, 15). Some women favored advice provided by a health professional, preferably with specialist training in preconception health, over online information, due to their greater credibility. This mirrors prior findings that women with diabetes value high-quality information relating to pregnancy (20, 21). However, we also found that, due to their perceived inaccessibility, participants commonly felt it was inappropriate to consult GPs for information retrievable online. This echoes McGowan et al.’s findings that men and women are reluctant to seek preconception care from a physician due to a perceived lack of availability and a view that this would be ‘wasting an appointment’ (25). Collectively, these findings suggest that an awareness of primary care capacity issues may drive some women to less credible but more accessible information sources, and highlight the trade-offs women may make when seeking reproductive health information.

Participants also reported that they did not want preconception health discussions with a health professional to be judgmental. This echoes prior findings that fears of being judged, labelled or lectured (19, 20) can impede the receipt of preconception care by women with diabetes, suggesting these findings may be generalizable to other reproductive-age populations. Women also favored positively-framed information that highlights the benefits of good preconception health rather than risks and harms. This replicates McGowan et al.’s (25) finding that participants were uncomfortable with messages that link parental preconception behaviors with adverse infant outcomes. Similarly, women in our study recommended emphasizing that adhering to all preconception health guidelines does not guarantee a healthy baby, to minimize the potential for self-blame for adverse outcomes. Participants also widely considered real people’s lived experiences to be more relatable and memorable than textbook, scientific information. This can be seen to reflect the tenets of Fisher’s narrative paradigm, that people draw on an innate narrative logic to assess new information, and that all meaningful communication happens via storytelling (52).

There were some notable tensions between the views expressed in this study and the epidemiological literature on preconception health. For instance, our finding that some women felt preconception health interventions should include support for preconception mental health contrasts with the lack of systematic reviews reporting associations between this factor and reproductive outcomes (4). This suggests that women may want the focus of preconception interventions to extend beyond risk factors that have currently demonstrable adverse health impacts to also encompass a broader range of social, mental health and well-being factors.

### 4.2 Implications for policy, practice and future research

Researchers, policymakers and practitioners should seek to develop and implement preconception interventions informed by the strategies we identified to enhance the appeal and acceptability of their delivery and content (16, 17). Our finding that women commonly considered GPs and practice nurses to be relatively trustworthy but inaccessible intervention delivery methods has implications for the English government’s aim for primary care professionals to have a role in supporting women’s preconception health (13). It suggests that to achieve this, strategies are needed to persuade women that seeking this support is an appropriate reason to see a GP or nurse. As primary care professionals have repeatedly reported capacity issues to be a major barrier to providing preconception care (27, 28), innovative, light-touch interventions may be required to build capacity for this. This could include raising the topic of preconception health in existing encounters relating to reproductive issues or women’s health, such as cervical smear tests and contraceptive counseling; participants considered this more acceptable than raising the topic ‘out of the blue’. If this approach is taken, future research should seek to identify the training and resources these professionals require to deliver this opportunistic preconception care in an acceptable way. An alternative interpretation of women’s reluctance to seek preconception care from primary care is that this further highlights a need to consider online and community-based opportunities for preconception health interventions (9, 31).

Our findings highlight complexities for preconception health interventions and trade-offs that may be needed to tackle these. The possibility that a focus on weight may deter some women from engaging in preconception interventions is one such challenge. Obesity is considered a major public health issue (53) and pre-pregnancy obesity is a risk factor for common adverse pregnancy outcomes including pre-eclampsia and gestational diabetes (4). On this basis, it is understandable that preconception health interventions should aim to reduce weight in mothers-to-be with obesity. However, policymakers and practitioners should consider the relative benefits of this approach against the risk of deterring these women from engaging in these interventions and potentially seeing improvements in other risk factors. They should be mindful of the stigma mothers with obesity already experience (54) and of concerns that medical practice draws on prevailing “*biomedical discourse*” to justify controlling women’s bodies (55). This highlights the potential merits of using ‘dark logic’ models in the early development of preconception health interventions, to explore the potential for unintended harmful effects (56). Future research should explore whether it may be more acceptable for these interventions to focus on weight-related behaviors, such as diet and exercise, rather than having explicit weight-based goals.

### 4.3 Limitations

Our study has some limitations relating to its sample and design. We recruited a balanced sample with respect to age and gravidity and the proportions of participants who were born outside the UK and had household incomes under £32,000 were in line with the national average (47, 49). However, the actual numbers of participants from these groups were small and university graduates were overrepresented (50). Our findings need to be interpreted with an awareness of this. Further, women who were considered likely to be distressed by pregnancy-related content were not included (31). A finding of this study was that preconception interventions need to be sensitive to the experiences of these women, and their exclusion means their views on how to ensure this were not reflected. Our use of a sequential explanatory mixed methods design, where we analyzed our quantitative data (31) before collecting our qualitative data, allowed us to qualitatively explore our survey’s findings and purposively recruit women from the general population. This overcame many of the limitations and potential bias of prior qualitative studies in this area, which used convenience samples (24, 25). However, it should be noted that only a minority of the women who received a questionnaire took part in our survey (31), which may have introduced a self-selection bias if participants’ experiences and views on preconception health were different to those who did not participate. Further, a sequential exploratory design would have allowed us to quantitatively assess the representativeness of the views participants expressed and the appropriateness of transferring our findings to different samples (156). This is a potential avenue for future research.

Additionally, the positioning of the first author as an outsider researcher may have affected the study’s knowledge production. This likely helped to avoid issues associated with insider research, including over-familiarity, taken-for-granted assumptions and the omission of details from participants’ narratives due to assumed common understanding and experience (57). Placing the participant in a relatively expert position, argued to be an ‘empowering experience’, can also be advantageous when studying marginalized individuals (58). Conversely, MD’s positioning as an outsider researcher may have meant there was information relating to the reproductive experiences of the interviewed women they did not feel comfortable sharing with a man (58).

## 5 Conclusion

Women favored preconception health interventions that ensure discretion, easy access and sensitivity, seek consent to receive the intervention, and provide trustworthy information that highlights the benefits of good preconception health rather than stigmatizing people for their weight, actions and reproductive choices. The use of visual media, personalization, interesting and unfamiliar facts and simple information with signposting to more detailed information were also viewed favorably. Future research should seek to develop and implement preconception health interventions informed by these findings, and should explore the potential for these interventions to have unintended harmful effects.

## Data availability statement

The raw data supporting the conclusions of this article will be made available by the authors, without undue reservation.

## Ethics statement

The studies involving humans were approved by South West – Frenchay Research Ethics Committee. The studies were conducted in accordance with the local legislation and institutional requirements. The participants provided their written informed consent to participate in this study.

## Author contributions

MD: Conceptualization, Data curation, Formal analysis, Investigation, Methodology, Project administration, Validation, Writing – original draft, Writing – review & editing, Funding acquisition. RK: Conceptualization, Funding acquisition, Supervision, Writing – review & editing. JW: Conceptualization, Funding acquisition, Supervision, Writing – review & editing. JS: Conceptualization, Investigation, Supervision, Writing – review & editing.
