# Correction: Women's views on content and delivery methods for interventions to improve preconception health: a qualitative exploration

**DOI:** 10.3389/fpubh.2026.1858842

**Published:** 2026-05-07

**Authors:** Michael P. Daly, Ruth R. Kipping, James White, Juli Sanders

**Affiliations:** 1 Department of Population Health Sciences, Bristol Medical School, University of Bristol, Bristol, United Kingdom; 2 School of Medicine, Cardiff University, Cardiff, United Kingdom; 3 School of Healthcare Sciences, Cardiff University, Cardiff, United Kingdom

**Keywords:** preconception care, preconception health, women's health, intervention development, intervention design, qualitative, framework analysis, sequential exploratory design

An incorrect grant number was provided for the GW4 BioMed DTP. The grant number was displayed as “MR/N0137941/1”. The correct number is “MR/N013794/1.”

The original version of this article has been updated.

