# Supplementary material for: Women’s views on content and delivery methods for interventions to improve preconception health: a qualitative exploration
Source: Front Public Health. 2024 Feb 21;12:1303953. doi: 10.3389/fpubh.2024.1303953 (PMC10915026; doi:10.3389/fpubh.2024.1303953)
Supplement: Supplementary file 1 [file Table_1.DOCX]

Supplementary Material

Contents

[S1: Participant sampling criteria 1](#_Toc146727875)

[S2: Interview topic guide 2](#_Toc146727876)

[S3: Deductive codes used in the qualitative analysis 9](#_Toc146727877)

[S4: Quality assurance measures to ensure data trustworthiness 10](#_Toc146727878)

[S5: Participant attitudes toward preconception health 11](#_Toc146727879)

[S6: Summary points for each intervention delivery method explored in the interviews 12](#_Toc146727880)

[References 20](#_Toc146727881)

# S1: Participant sampling criteria

|  | **Survey variable** | **Dichotomy** |
| --- | --- | --- |
| **Primary criteria** | Age  Ever pregnant  Household income  Knowledge of maternal preconception risk factors | 18-29 years versus 30-48 years  Previously pregnant versus never pregnant  <£32,000 vs ≥£32,000 per year  Listed ≤2 versus ≥3 of the assessed maternal preconception risk factors |
| **Secondary criteria** | Ethnicity  Country of birth  Pregnancy intentions  Intervention delivery method acceptability  All five attitudinal variables (interest, intentions, self-efficacy, perceived awareness & importance) | White ethnicity versus minority ethnicity  Born in the UK versus born outside of the UK  Wanting a future pregnancy versus unsure or definitely not/unable to get pregnant  Positive versus neutral or negative responses for each discussed intervention method  Positive versus neutral or negative responses for each of these variables |

# S2: Interview topic guide

**Equipment**

• Encrypted audio-recorder (Olympus Digital Voice Recorder DS-3400)

• Telephone device (Olympus TP8)

• Spare batteries

• Pens and paper

• Consent form

• £20 Love2shop voucher and stamped envelope

**Introduction**

• Hello [name], this is [Michael/Ruth/Julia] from the University of Bristol/Cardiff University speaking, how are you? Have you had a chance to look at the Information Sheet (and Consent Form) I sent you with my email the other day?

- *If yes to the above*: Great – is it still convenient for you to do the interview now? It will take

up to an hour

• *If yes*: Great - Thank you so much for sparing your time.

• So before starting the interview, I’m just going to talk a bit about the study, which is a part of a wider doctoral project/a doctoral project I’m supervising/helping with. I’m then going to record your consent to take part

• So firstly, just to give a bit of background on the study, we want to find out what women know and think about health before pregnancy, which is sometimes called preconception or pre-pregnancy health. We also want to explore women’s views on ways of improving health before pregnancy in the UK. This is to give us a better idea about what information may be helpful for women/couples before pregnancy and how best to give it

• This interview will just be a discussion about these topics and some of your survey answers, rather than another questionnaire. There are no wrong or right answers, and you don’t have to answer all of the questions if you don’t want to.

- Feel free to ask questions or ask to stop the interview at any time.

• Anything that you tell me is confidential and won’t be linked to you – when the interview is typed up, any specific names or places you mention will be removed, so that only anonymised quotes will be used in the reports, publications or teaching materials that come from this study. The only reason I would need to break confidentiality would be if you say something where I am concerned about harm to you or someone else.

• When I start the voice recorder, I’m going to read out each statement on the version of the study consent form (you received by email/in the post), and if you could please confirm that you’re happy with each statement I will sign them for you on your behalf. Sorry that this might seem a bit formal and lengthy, but this is just something we have to do as part of the University ethics regulations.

• Do you have any questions before I start the recording? If it’s ok, I will start recording now.

START RECORDING

- *State interviewer name, participant name, participant ID number and date/time of interview*
- *Read out each statement on the consent form. Ask participant to say ‘Yes’ after each statement if they are happy with the statement.*
- *Explain to the participant that you are now going to stop the audio-recording, and re-start recording for the main part of the interview. Explain that this is so that their name can be kept separate to the rest of the interview*

STOP ABOVE RECORDING & START NEW RECORDING

- *State interviewer name, participant ID and date/time of interview (do NOT state participant name)*

**Background and personal circumstances**

*Aim: To highlight any key background factors that might influence their views*

- Just to provide some background information, please could you tell me whether you have any experience of pregnancy?
- Have you ever been pregnant?
  - Do you remember making any health or lifestyle changes before your pregnancy(ies)?
    - Probe: Can you remember why you made those changes?

• Do you think you might have any more children?

1. **Preconception health knowledge, attitudes and behaviours**

*Aim: To explore the participant’s knowledge of, attitudes (interest, intentions, perceived awareness and importance) towards and behaviours around preconception health*

Introduction: Now I’ll be asking you about your views on health before pregnancy. These next few questions are based on some of your answers in the survey, so I’ll be asking you whether you would still give the same answers and if you could say a bit more about them

***Perceived importance***

- You mentioned on your questionnaire that you (*Strongly agreed /Agree /Neither agreed nor disagreed /Disagreed /Strongly disagreed*) that a woman’s health and lifestyle before pregnancy can affect the health of her and her baby during and after pregnancy.
  - Would you still say this? Could you tell me a bit about why you gave that answer?
  - Would you say the same/give a different answer for a woman’s health and lifestyle during pregnancy?
    - Probe: Do you think a woman’s health during pregnancy might be any more or less important than her health just before pregnancy? Could you tell me about why you would say this?
    - Fertility: What about the health of the woman and her baby during & after pregnancy – do you think there might be any links between preconception health and those things?
  - As I mentioned earlier, health before pregnancy is called different things by different people, such as preconception health or pre-pregnancy health. What would you prefer to call it?

***Perceived awareness.***

The questionnaire also asked you to rate how aware you feel you are of the positive behaviours and other actions women can take before pregnancy to help to have a healthy pregnancy and a healthy baby – you said you were (*Very/ Moderately/ Slightly/ Not at all*) aware

- Could you tell me a bit about why you gave that answer?
- *Low awareness score*: What might help you to feel more aware of these things?
- *High awareness score*: What information sources have made you more aware of these?

***Interest***

- You said on your questionnaire that you were (*Very/ Moderately/ Slightly/ Not at all*) interested in knowing more about pre-pregnancy health.
  - Where does that interest come from?
  - Do you think anything would make you more or less interested in this information?
    - Probe: Can you think of anything that might affect other women’s interest in this?

*Where a participant answered “Strongly agree” or “Agree” to the Perceived importance item but gave a low likelihood rating for the Intentions item:*

***Intentions***

- One of the other questions on the questionnaire asked you to imagine that you were planning to become pregnant within 6 months. You were then asked how likely it was that you would make any lifestyle changes during these 6 months, in preparation for pregnancy. You answered (*Very likely /Quite likely /Neither likely nor unlikely /Quite unlikely /Very unlikely*).
  - Could you tell me why you gave that answer?
    - What sort of changes had you in mind?
    - With those changes, would you make them/do you think it’s important to make them before pregnancy, or would it be okay to wait until you become pregnant to make them? Why’s that?
  - What might affect the chances of you making such changes?
    - Probe: In other words, what might help or prevent you from making them?
    - Probe: Why might some women make changes in the time before pregnancy while other women wouldn’t?

**2. Methods to improve preconception health**

*Aim: To explore participants’ views on different methods to improve preconception health in the UK and how to maximise the acceptability and appeal of these methods*

Introduction: Now I’ll be asking you about your views on different ways of improving health before pregnancy in the UK

***Participant-suggested methods*** ***to improve preconception health***

- Can you think of any ways women in the UK could be helped or supported to improve their health before pregnancy?
- Do you think there’s a need for this help and support? Do you think other women would say the same?
  - *For women who evidenced lower knowledge of preconception health risk factors in the survey*: Some evidence suggests that things like [taking folic acid, BMI, physical activity, diet…etc…] in the time before pregnancy *might* affect whether a woman has a healthy pregnancy and baby. How might women be helped and supported to make positive changes in these areas before pregnancy?
  - *For women who evidenced greater knowledge in the survey*: In your questionnaire, you mentioned things like [taking folic acid, exercising.. etc.. ]. Some evidence suggests these things [as well as…the amount of time between pregnancies.. etc.. ] *might* affect whether a woman has a healthy pregnancy and baby. How might women be helped and supported to make positive changes in these areas, before pregnancy?
  - Probe: What might make it easier or more difficult for them to make changes in these areas?
  - Probe: These might be things like policies [*explain as ‘institutional changes’/changes at a national level, if needed*], advice, information or services
    - Probe: Examples might be things like adding folic acid to food products, or inflating the cost of alcohol to reduce consumption, which has been done in countries like Canada.
    - What do you think of those suggestions -are there any others you can think of that might be helpful or unhelpful?

***Methods to improve preconception health derived from the literature***

- In the survey, women were asked how comfortable they would be discussing health before pregnancy and their personal pregnancy plans with a number of different people.
- I’d like to discuss some of the most popular options with you in turn. The first/next group of people were [*GPs/ nurse practitioners/ pharmacists* and you said you would be *(Very comfortable /Somewhat comfortable /Neither comfortable nor uncomfortable /Somewhat uncomfortable /Very uncomfortable)* discussing health before pregnancy and your personal pregnancy plans with these – could you tell me more about why you thought that.
- What might be the issues around discussing health before pregnancy and your personal pregnancy plans with these people? Are there any particular aspects of your health or lifestyle you would personally want to talk to health professionals about before pregnancy?
- Would you be comfortable discussing these things with [x] at any appointment/encounter, or just at particular appointments/encounters?
  - Would this discussion need to be started by you, or are there times/ways they could ask if you’d be interested in discussing these things that might be appropriate?
- In the survey, we also asked women to rate how acceptable it would be (*to them*) if information about health before pregnancy was made available in a number of different places.
- Again, I’d like to discuss some of the most popular options with you in turn. The first/next was [*including this information with pregnancy tests/ including it in health education in schools/ providing it through social media/ providing it by personal text or email, for example from your GP* and you said that would be (*Very acceptable /Somewhat acceptable /Neither acceptable nor unacceptable /Somewhat unacceptable /Very unacceptable*) – could you tell me more about why you thought that.
- What might help to make providing information about health before pregnancy in this way more acceptable?
- “ “ “ more engaging and appealing?
- What about the information itself – what would be the ‘best way’ of wording it?
- Are there any other methods to help promote or improve pre-pregnancy/preconception health you think should be considered?

**End**

- We’re just coming to the end of the interview now [name] - Is there anything we haven’t covered that you’d like to add?
- That’s all the questions I have for you today. Do you have any questions for me?
- I will stop recording the interview now.

STOP RECORDING

- [Name] thank you so much for your time - I really appreciate you sharing your views with me.

*Reassure them about confidentiality and anonymity*

- (If not already provided) Please could you let me know a postal address so that I can send you a £20 Love2shop voucher to thank you for your time. Great – thank you. I’ll also send you a page with this that provides information on charities and organisations that help women with some of the topics we discussed – we send this to all interviewees.
- Thank you. Lastly, would you like to be informed of the outcomes and findings of this research?

*If yes*: Is it ok to use the email address you provided with your questionnaire for this?

- It was lovely speaking to you, thank you again, enjoy the rest of your day

# S3: Deductive codes used in the qualitative analysis

1. General practitioner/Nurse practitioner/Pharmacist *(discussing preconception health and pregnancy plans with a “)*

a. : advantage

b. : disadvantage

c. : acceptability

d. : discussion timing

e. : discussion content/wording

2. Pregnancy tests/Health education in schools/Personal text or email/Social media *(providing preconception health information with/through/by)*

a. : advantage

b. : disadvantage

c. : acceptability

d. : appeal/engagement

e. : information content

# S4: Quality assurance measures to ensure data trustworthiness

| **Data trustworthiness criteria (1)** | **Quality assurance measures (to meet these criteria)** |
| --- | --- |
| Credibility *(participants’ views are well represented by the research (2))* | - Multiple participants were interviewed, previous research informed the development of the deductive codes, and the themes represented the views of multiple participants. - Co-investigator feedback on the first author’s interpretations of the raw data and developing themes. - Reflexivity review to identify how our positionality shaped the research and account for this (3, 4). |
| Dependability *(the findings are consistent and reproducible (2))* | - Use of the framework analysis method; this method’s use of ‘audit trails’ to map analytic progress provides analytic transparency (5, 6). - Reflexivity review and memos recording analytic decisions and reasonings, to improve the transparency of the research process (7). |
| Transferability *(the findings can be generalised or extrapolated to other contexts (2))* | - Maximum variation purposive sampling approach with sampling criteria which represented ‘salient characteristics’ (8, 9). - Detailed description of the study, its context, and the participant sample, to allow readers to draw conclusions about transferability. - Discussion of the study sample’s limitations. |
| Confirmability *(the findings are demonstrably drawn from the data (2))* | - Review of the interview transcripts to ensure candidate themes captured participants’ verbatim views. - Use of participant quotes to support the presented themes. |

# S5: Participant attitudes toward preconception health*

| **Attitudinal variable** | **Response categories** | **N** | **%** |
| --- | --- | --- | --- |
| Perceived awareness of maternal preconception risk factors | Not aware at all  Slightly aware  Moderately aware  Very aware | 1  6  5  8 | 5  30  25  40 |
| Perceived importance of preconception health | Strongly disagree  Disagree  Neither agree nor disagree  Agree  Strongly agree | 1  1  1  5  12 | 1  1  1  5  12 |
| Interest in knowing more about preconception health | Not at all interested  Slightly interested  Moderately interested  Very interested | 2  7  3  8 | 10  35  15  40 |
| Preconceptional self-efficacy | Strongly disagree  Disagree  Neither agree nor disagree  Agree  Strongly agree | 0  0  4  5  11 | 0  0  2  25  55 |
| Preconception behaviour change intentions | Very unlikely  Quite unlikely  Neither likely nor unlikely  Likely  Very likely | 2  2  0  5  11 | 10  10  0  25  55 |

Legend: * The data in this table correspond to the interviewed participants’ questionnaire

responses in our prior survey study (10)

# S6: Summary points for each intervention delivery method explored in the interviews

| **Medium** | **Summary points** |
| --- | --- |
| **General practitioner (GP)** | ***Advantages:***   - Lack of judgement and coercion - Can be trusted not to share personal information - Are “*the real medical practitioners*” [P.02] who “*have obstetric experience*” [P.19] and generally give patients “*information they can trust*” [P.10] - Are “*good at diagnosing things*” [P.10] and helping people ascertain their “*body's ready*” for pregnancy [P.07]   ***Disadvantages:***   - Lack of availability: it is difficult to get an appointment and there is not enough time within appointments to have a “*proper conversation*” [P.03] - Perceived lack of support for reproductive issues - View that care from a GP *“varies a lot*” [P.10] - A GP’s knowledge is “*spread over so many things*” [P.17] and someone who is more “*specialised*” [P.13] in preconception health might know more - Some felt they would be “*very uncomfortable*” [P.09] or “*very anxious*” [P.04] discussing preconception health with a GP due to previous negative experiences relating to their weight - Some participants were uncertain about whether they would want to discuss “*basic lifestyle changes*” [P.06] with a GP   ***Suggestions:***   - Seeing a doctor *“for the sake of seeing a doctor*” when trying to get pregnant should become “*normalised”* [P.16] - GPs should “*keep their personal opinions to themselves*” [P.10] and not pressure patients to do anything *“they don't want*” [P.07] |
| **Medium** | **Summary points** |
| **Nurse practitioner** | ***Advantages:***   - It was generally felt that nurses have “*more time*” [P.03] and are “*a lot easier to see*” [P.09] than a GP, and that additional topics, such as preconception health, can be raised in nurse consultations alongside other care - Some felt nurses have more “*hands-on experience of how things have affected people*” [P.10] and are “*a bit more down-to-earth*” [P.12] than GPs, whose vast knowledge can be intimidating - As nurses are “*more patient-facing”* they may have a greater ability to “*make someone feel comfortable to open up to talk about pre-pregnancy experiences*” [P.15] - Like GPs, nurses are “*experts to a degree*” and would “*know their limits in terms of their knowledge*” [P.11]   ***Disadvantages:***   - Some felt that, like GPs, they wouldn't want to *“waste*” a nurse’s time on something they *“didn't feel was necessary*” [P.05] as nurses are also “*so busy*” [P.12] - Some felt that nurses’ more “*practical*” [P.03] skill set means they are a less acceptable source for health guidance   ***Other views:***   - Some felt the relative acceptability of a nurse versus a GP would depend on which of these has the most specialist knowledge relating to preconception health |
| **Medium** | **Summary points** |
| **Pharmacist** | ***Advantages:***   - Widely considered to have better availability than GPs - Can allow patients to remain somewhat anonymous - Participants commonly saw pharmacists as the “*medicine person*” [P.08] with whom they would feel comfortable discussing preconception supplements and the impacts of “*other medication[s]*” [P.01] - Some felt that pharmacists are “*really willing to give information and give advice*” and have a “*duty of care*” to do so [P.03], and that they go “*above and beyond*” and can be “*really helpful*” [P.06]   ***Disadvantages:***   - Enabling discretion and privacy may be an issue due to the public nature of (most) patient discussions with a pharmacist - Some felt pharmacists’ skill set is “*quite specific*" [P.19] and that, unlike supplements and medication, they would not seek their advice on “*lifestyle changes*” [P.09] - Some participants reported negative experiences with pharmacists who were “*not very helpful*” and just told them to “*go to the doctor*” [P.04]   ***Suggestions:***   - It would be more appropriate for pharmacists to discuss preconception health with patients if they had “*time to take [them] aside*” [P.03] and consult them in a private room. - There should be an option to book this private discussion online, as publicly requesting this may be undesirable for some   ***Other views:***   - Some felt it would only be acceptable for a pharmacist to raise one’s pregnancy plans and preconception health “*if they’re trained*” to do so [P.06] or if their pharmacy has “*a specific interest in maternal health*” [P.19] |
| **Medium** | **Summary points** |
| **Primary care practitioner**  **(general)** | ***Advantages:***   - Some expressed that receiving advice from a health professional is better than getting information online, as you can be more sure you have "*got the right information*" [P.06] - Participants also valued the “*personal touch*” of a health professional-initiated conversation over, for instance, “*a leaflet in a GP surgery”* [P.12]   ***Disadvantages:***   - Participants reported that they only contact these professionals about illnesses and medical emergencies, as they lack the time to merely provide “*information*” [P.01] about issues like preconception health - Some felt health professionals raising the topic of preconception health might be seen to disregard the wishes of women who don’t want children and “*panic*” [P.11] those who have little time remaining to become pregnant   ***Suggestions:***   - Some participants suggested it would be more acceptable for a health professional to raise preconception health and pregnancy intentions in patient consultations related to reproductive issues or female “*anatomy*” [P.11] - Others felt this would only be appropriate if patients had mentioned a product or issue related to pregnancy, had shown an interest in preconception health information, or had been asked about or indicated their pregnancy intentions - Suggested ways that these professionals could advertise preconception health information and services so as to enable patients to choose whether they engage with these included “*a welcome pack*” [P.05] for new registrations and promotional materials such as posters - Some women conveyed a preference for speaking to a female health professional about preconception health and their pregnancy plans. A view that professionals who are “*female, or a mum*” are more likely to go “*to the length of finding out*” [P.01] about these issues was also noted - Some expressed that they would be more comfortable discussing preconception health with a professional they have a personal relationship with   ***Others views:***   - Many expressed that their trust in health professionals’ advice would depend on the perceived skill set and knowledge remit of that professional. - A few participants described how it was “*not reasonable*” that “*so much of [their] medical care*” [P.09] had revolved around their weight, that the way they have been spoken to by health professionals about their weight has been “*awful*” [P.04], and that these professionals are overly focused on advising weight loss and lack the ability to support patients beyond this - Others felt health professionals are overly focused on pregnancy prevention with young women, relative to pregnancy education - Some felt it should “*be standard*” [P.03] for practitioners to ask questions like “*do you feel well equipped with the knowledge to have a successful pregnancy?*” [P.03] as “*a lot of people won't… want to admit that they don't know this*” information [P.03] |
| **Medium** | **Summary points** |
| **Personal text/email *(e.g., from a general practice)*** | ***Advantages:***   - A convenient and “*discreet way*” [P.18] of providing information   ***Disadvantages:***   - May not be accessible to some people, such as “*older women*” [P.15] who don’t have phones and email   ***Suggestions:***   - Only send these to patients “*from the age of eighteen*” [P.17] - Personalise the provided information through, for instance, using a patient’s name or following on from a relevant conversation - To enable patient choice, use a scoping message asking “‘*are you happy to receive emails on this topic?*’” [P.13], a “*click to find out more*” [P.14] or “*opt-in*” [P.03] option, or ask patients “‘*Is it okay if I send you this stuff?*’” [P.13] at their appointments - Include a content warning (e.g., at the “*very top of the email*” [P.05]) so that recipients can make an informed decision about whether to “*delete or to read*” [P.05] the information |
| **Pregnancy tests** | ***Advantages:***   - “Q*uite private”* [P.13] as “*nobody needs to know*” [P.12] you’re engaging with the provided information - “*Useful*” [P.16] way of getting this information to people who are actively trying to conceive and have an immediate need for it   ***Disadvantages:***   - Some felt this delivery method isn't “*of benefit*" [P.02] if the recipient is already pregnant and “*ideally you'd want to have it before you saw the test”* [P.14]. - Others felt this approach involves a problematic assumption “*that people who are taking pregnancy tests are wanting to conceive*” [P.08] unless information on how *“to prevent*” [P.08] pregnancy would also be included   ***Suggestions:***   - Place the information inside the pregnancy test packaging rather than having a vendor publicly provide this over a counter [P.18] - Provide informational leaflets *by* rather than *in* pregnancy test boxes [P.09], use this leaflet to merely signpost to “*where [one] can look*” [P.17] for information, and have the information in a separate envelope so that recipients can choose to “*discard it*” [P.14] without having to read it - Include this information with “*more expensive”* pregnancy tests as those are the ones that *“people tend to do when they're trying to conceive*” [P.09] |
| **Medium** | **Summary points** |
| **Social media** | ***Advantages:***   - A resource that people can “*access in the privacy of their own homes*” [P.19] - Not as invasive as having “*information sent straight to your phone*” [P.10] as people choose to use social media and have some control over the content they want to see and engage with on it. - An “*accessible*” [P.13] method where information can be accessed in one’s own time and passive information receipt can lead to real engagement - Would be *“far-reaching*” [P.10] as “*almost everyone is on social media in one format or another*” [P.16], including “*people who might not be able to access information elsewhere*”[P.19] - Could enable users to “*find support with other women, or other people*” [P.09]   ***Disadvantages:***   - Information provided through social media may get “*lost amongst all of the reams of misinformation*” [P.18] social media users are exposed to, as it is difficult to discern legitimate information from this misinformation   ***Suggestions:***   - Privacy-enhancing suggestions included having information appear “*on your [news] feed and not your friends*’” [P.05] and having the option to “*sign up to groups*” where the information is “*visible just to you*” [P.05] so that recipients can discreetly respond to this - “*Reputable ways*” [P.03] of delivering preconception advice on social media included partnerships between respected institutions and subject experts and the use of “*.org*” websites [P.07], “*peer-reviewed*” literature [P.18], professional moderators, and recommendations from established health organisations such as the National Health Service - Visual media can turn “*a very boring, factual [social media] post into something that catches people's attention*” [P.13] and it’s “*almost not even worth*” [P.03] putting information on social media without this   ***Other views:***   - Some felt targeted social media advertisements would be acceptable as: these “*tend to be related to either [online] searches or conversations*” [P.01]; social media algorithms are “*very for that person*” [P.09]; and users have the option to say they don’t want to see similar information in the future - Others felt the use of targeted advertisements would invade the privacy of those who have experienced adverse outcomes, and that the imperfection of algorithms means there would be issues with the targeting. They thus felt information should only be provided to those who have explicitly sought it out - A view that targeted social media advertisements, based on relevant internet searches, are more likely to reach women who are already looking to improve their preconception health and thus disadvantage women with fewer resources was also noted |
| **Medium** | **Summary points** |
| **School health education** | ***Advantages:***   - Some felt this method would deliver information at a relevant time to women who become pregnant during or soon after school, who often have the greatest need for this information and may lack other means of receiving it. - Others felt that, whilst the teenage years are not the “*ideal*” time to receive this information, the advantage of being able to “*catch*” this group “*in one place*” [P.17] outweighs that issue - Women are less likely to have experienced an adverse pregnancy outcome by this point in their lives and information learnt in school “*stays with you for quite a long time*” [P.09]   ***Suggestions:***   - It may be necessary to relay preconception health information in “*a fun way*” [P.09] to children (e.g., using a game or a children’s book) - Pupils should have a way of accessing this information discreetly (e.g., “*having good links to information sites*” [P.10] on school websites) - Some felt teachers are best placed to provide this information as they have “*rapport*” [P.06] with their students and this would help to create a culture where students feel they can ask teachers questions *“outside of sex education without either the teacher or the student being scared of it.*” [P.10] - Others felt that a health professional delivering this information in schools might have “*more authority”* and would be better “*able to manage [any] questions”* and *“make it a bit more engaging”* [P.08]. Some also felt this would mean students know *“they're being taught the right thing”* and it would *“be more of a memorable experience”* [P.09] - Participants also suggested delivering this information as a seminar or “*open talk*” [P.08], or across multiple lessons where the information “*develops*” [P.10] as the pupil gets older   ***Other views:***   - All participants agreed that providing at least *some* information about preconception health to school-aged pupils would be appropriate - Specifically, participants suggested teaching pupils how to “*look after themselves and keep themselves healthy*” [P.12] and informing them of relevant “*services*” [P.05] and “*where to access [relevant] information*” [P.08]. They also suggested highlighting that the preconception period is a unique phase of the pregnancy journey and highlighting the “*problems that can occur*” [P.04] during this period, such as infertility - Views on which age range of children and young people it would be appropriate to provide this information to, and the depth of information that should be provided, were more nuanced - Some felt that this information should be communicated to pupils - particularly older teenagers - in the same way it would to adults, as “*making everything very kiddy and funky*” [P.06] can be off-putting and impair understanding |
| **Medium** | **Summary points** |
| **School health education**  ***(continued)*** | - Others felt it may be better for the focus of this information to be “*women’s health*” [P.03] or the importance of preconception health more generally, as topics like babies and pregnancy may be off-putting for this cohort - For younger children and teenagers, it was widely expressed that providing less information and avoiding “*heavy*” [P.01] topics such as risk factors for miscarriage and stillbirth may be more appropriate, due to concerns that these pupils are not mature enough to put this information into context and that this may encourage young motherhood - Others suggested it would be more appropriate to only provide this information - or to provide more information - to older teenagers, with some suggesting that colleges and universities may be better settings than schools as recipients may be more “*receptive*” [P.03] at that point in their lives |

# References

1. Lincoln YS, Guba EG. But is it rigorous? Trustworthiness and authenticity in naturalistic evaluation. New Directions for Program Evaluation. (1986) 1986:73-84. doi: 10.1002/ev.1427

2. Nowell LS, Norris JM, White DE, Moules NJ. Thematic analysis: Striving to meet the trustworthiness criteria. International Journal of Qualitative Methods. (2017) 16:1-13. doi: 10.1177/1609406917733847

3. Cutcliffe JR. Reconsidering reflexivity: Introducing the case for intellectual entrepreneurship. Qualitative Health Research. (2003) 13:136-48. doi: 10.1177/1049732302239416

4. Braun V, Clarke V. Thematic Analysis: A Practical Guide. First ed. London: SAGE Publications (2021). 376 p.

5. Gale NK, Heath G, Cameron E, Rashid S, Redwood S. Using the framework method for the analysis of qualitative data in multi-disciplinary health research. BMC Medical Research Methodology. (2013) 13:1-8. doi: 10.1186/1471-2288-13-117

6. Ritchie J, Spencer L. Qualitative data analysis for applied policy research. In: Bryman A, Burgess R, editors. Analyzing qualitative data. London: Routledge (1994). p. 187-208.

7. Pillow W. Confession, catharsis, or cure? Rethinking the uses of reflexivity as methodological power in qualitative research. International Journal of Qualitative Studies in Education. (2003) 16:175-96. doi: 10.1080/0951839032000060635

8. Ritchie J, Lewis J, Nicholls CM, Ormston R. Qualitative research practice: A guide for social science students and researchers. Second ed. London: SAGE Publications (2013). 456 p.

9. Spencer L, Ritchie J, Lewis J, Dillon L. Quality in qualitative evaluation: a framework for assessing research evidence (2004). Available from <https://www.gov.uk/government/publications/government-social-research-framework-for-assessing-research-evidence>. [Accessed 24 June 2022].

10. Daly MP, White J, Sanders J, Kipping RR. Women’s knowledge, attitudes and views of preconception health and intervention delivery methods: a cross-sectional survey. BMC Pregnancy and Childbirth. (2022) 22:729. doi: 10.1186/s12884-022-05058-3
